# Association with amount of registration and outcome in pediatric severe trauma patients

**DOI:** 10.1186/cc14613

**Published:** 2015-03-16

**Authors:** S Ohnishi, N Saito, T Yagi, Y Konda, Y Hara, H Matsumoto

**Affiliations:** 1 Nippon Medical School Chiba Hokusoh Hospital, Chiba, Japan

## Introduction

Unexpected trauma is a one of the most major causes of death for children in the world. However, pediatric severe trauma patients are rare and scattered. Although there is a strong association between patient's volume of trauma center volume and outcomes in adults, such a report is less in children. In this study, we aimed to clarify the relationship of the amount of registration and outcome in pediatric trauma patients.

## Methods

This retrospective study analyzed data of the Japan Trauma Data Bank from 2004 to 2012. We registered pediatric patients aged younger than 12 years with severe multiple or torso trauma patients (maximum Abbreviated Injury Scale score ≥3 or Injury Severity Score ≥9, and excluded patients with cardiopulmonary arrest on arrival, burn, isolated head or limb injury, lack of data). We divided the facilities into six groups according to every 10 patients registered and compared mortality between each group.

## Results

A total of 1,015 patients from 105 facilities were included in the study. Number of registrations: 0 to 10 patients: 673 patients/68 facilities, 11 to 20: 381/28, 21 to 30: 141/6, 31 to 40: 101/3, 41 to 50: 45/1, 51 to 60: 58/1. Victims of blunt trauma accounted for 98.1%. Median age was 7 (interquartile range: 5 to 10) years, median injury severity score (ISS) was 17 (10 to 26). Multivariate analysis adjusted for age, revised trauma score and ISS revealed that the amount of registration (every 10 patients) was independently associated with survival (odds ratio: 1.55, 95% confidence interval: 1.06 to 2.26, *P *= 0.023).

## Conclusion

There was strong association between amount of registration and outcome.

